# Surfactant-Enhanced Cleaning Solutions for Ceramic Membranes: A Comparative Study on Humic Acid and BSA Fouling

**DOI:** 10.3390/membranes15030073

**Published:** 2025-03-02

**Authors:** Navneet Kallapalli, Onita D. Basu

**Affiliations:** Department of Civil and Environmental Engineering, Carleton University, 1125 Colonel by Drive, Ottawa, ON K1S 5B6, Canada; navneetkallapalli@cmail.carleton.ca

**Keywords:** NOM fouling, surfactant-based cleaning, fouling resistance, chemically enhanced backwash, ceramic membranes, drinking water

## Abstract

Control of natural organic matter (NOM) reversible and irreversible fouling with ceramic membranes for drinking water applications with chemically enhanced backwash (CEB) protocols is limited. This research examines the efficiency of various chemical combinations with non-ionic surfactants to control the NOM fouling caused by humic acid (HA) and protein foulants. Two commercially available non-ionic surfactants, Tween 80 and Triton X100, combined with conventional cleaning solutions, were analyzed with respect to membrane fouling and cleaning using the resistance in series (RIS) model, membrane permeability, carbon mass balance, and contact angle measurements. The results demonstrated that in all cases, CEB outperformed hydraulic backwashing; in addition, the inclusion of surfactants demonstrated enhanced the fouling control with protein foulants more than humic acid. The transmembrane pressure (TMP) with surfactant CEB was controlled to within a range of 83–105 kPa compared to hydraulic backwash at approx. 128 kPa for HA and BSA. The carbon mass balance analysis indicates that Tween 80 surfactant-based CEB demonstrated effective fouling control, leaving only 20% irreversible fouling with HA and 30% with BSA while the hydraulic backwash resulted in 57% irreversible fouling of carbon on the membrane for HA and BSA.

## 1. Introduction

Membrane applications in water treatment have increased drastically in the global market due to advancements in materials and process improvements. Low-pressure membranes (microfiltration (MF) and ultrafiltration (UF)) have experienced widespread acceptance for municipal and industrial water treatment alongside conventional water treatment methods [1,2]. In the low-pressure membrane market, polymeric membranes dominate within water treatment but have shorter life spans (approx. 5–10 years) due to material degradation from exposure to conventional cleaning solutions [3,4]. Ceramic membranes represent an alternative choice for consideration due to their reported higher chemical, mechanical and thermal tolerances, better pore-size distribution, higher fluxes at lower pressures, and a longer effective lifespan offering greater functional advantages compared to polymeric membranes [5,6]. Research into ceramic membranes for water treatment applications is critical to ensure transition into utility systems with ease of implementation.

Control of reversible and irreversible fouling is critical for the operation of membrane systems. Deposition of natural organic matter (NOM) and other substances onto the surface of the membrane, which can be removed effectively through hydraulic backwashing, refers to reversible fouling. On the other hand, irreversible fouling refers to the adsorption or pore plugging of NOM and other substances either within the pores or on the surface of the membrane, which cannot be eliminated by hydraulic backwashing [1,7].

Chemical cleaning is primarily aimed at mitigating the loss in permeability from irreversible fouling and restoring the membrane flux. The membrane’s compatibility and feed characteristics are among the factors that determine what type of chemical solution should be used [8]. Acid, alkali, oxidant, surfactant, and metal chelating agents are commonly employed cleaning agents [8,9,10]. Sodium hypochlorite (NaOCl) and sodium hydroxide (NaOH) are the most commonly used chemicals for preserving and restoring membrane flux [11]. These chemical solutions remove NOM fouling through oxidation, hydrolysis, and solubilization [8]. NaOCl facilitates the breakdown of NOM functional groups into carboxyl and aldehyde groups, hence simplifying their removal from the surface, and sodium hydroxide (NaOH) has the ability to dissolve organic compounds such as proteins and polysaccharides [6,12].

Cleaning of membranes is primarily performed by using clean-in-place (CIP) protocols, which involve soaking a membrane in a chemical solution for 30 min to several hours, generally followed by solution circulation and subsequent flushing [1,13,14]. In place of CIP, chemically enhanced backwash (CEB) protocols may be utilized to help control membrane fouling and delay the need for a CIP. CEB protocols employ the addition of lower concentrations of chemicals than a CIP process into a backwash cycle in place of the standard hydraulic backwash and can alleviate membrane fouling without removing the membrane from service [12,15]. Research into CEB protocols are somewhat limited and have been focused on more heavily within the polymeric fields [12,16,17,18].

In addition to traditional cleaning solutions, surfactants present a promising alternative for enhanced cleaning efficiency, versatility, and cost-effectiveness [19,20]. Surfactants form micelles at their critical micelle concentration (CMC) that abridge hydrophobic foulants, aiding their removal. These surfactants, combined with NaOH and NaOCl, may enhance cleaning by improving penetration within the foulant layer and facilitating chemical reactions that clean the membrane. Additionally, surfactants cause temporary changes to the characteristics of the membrane surface, reducing the forces that cause adhesion and limiting the reattachment of substances that cause fouling following the cleaning process [21,22,23]. Previous studies have shown that combining surfactants with traditional cleaning agents improves cleaning performance and membrane longevity [16,24,25,26]. Levitsky et al. (2012) observed that the CEB of the combined cleaning solution, i.e. Tween 20 (a non-ionic surfactant), NaOCl, and NaOH effectively restored water flux at low chemical concentrations, resulting in reduced protein fouling on a polymeric PES membrane and no evidence of membrane disintegration [16]. Gul et al. (2022) reported that a flat sheet PAN nanomembrane subject to a 24 h CIP soak with a binary solution of Triton + 5% NaOH demonstrated the highest flux recovery for the removal of engine oil wastewater compared to Triton or NaOH alone [7]. Overall, research on combined cleaning solutions with surfactants is primarily focused on polymeric membranes.

Among non-ionic surfactants, Tween (a polysorbate-based surfactant) and Triton (an octylphenol ethoxylate-based surfactant) demonstrate distinct cleaning mechanisms that affect their efficacy in the removal of membrane fouling. Tween 80, a hydrophilic surfactant with a high hydrophilic-lipophilic balance (HLB), enhances fouling removal by reducing surface tension, improving membrane wettability, and facilitating the detachment of hydrophobic NOM components [22,27]. Conversely, Triton X-100, with its relatively lower HLB, interacts more strongly with hydrophobic foulants such as proteins, aiding in their emulsification and dispersion [7,28]. These differences influence the cleaning performance of chemically enhanced backwashing when used in combination with NaOH and NaOCl. Understanding the specific interactions between these surfactants and NOM foulants, such as humic acid (HA) and bovine serum albumin (BSA), is crucial for optimizing membrane cleaning protocols.

Research on surfactant-based cleaning, specifically on natural organic matter (NOM) fouling with CEB, and ceramic membranes, remains scarce. Thus, this research investigates the beneficial effect of utilizing a CEB protocol with non-ionic surfactants and conventional cleaning solutions for the removal of NOM foulants from ceramic UF membranes. This research assesses critical parameters such as transmembrane pressure, RIS fouling index, TOC mass balances, and permeability recovery. The current work systematically investigates the interactions between non-ionic surfactants and ceramic membranes and aims to enhance the recovery of membrane fouling; it also aims to develop a more effective and sustainable cleaning approach for UF ceramic membranes.

## 2. Materials and Methods

**Model foulants:** NOM model foulants used in this study were hydrophobic NOM, humic acid (Sigma Aldrich, St. Louis, MO, USA lot# BCCB6671) at 5 mg C/L, and hydrophilic protein, bovine serum albumin (BSA) (Sigma Aldrich, St. Louis, MO, USA) at 5 mg C/L. As reported in previous studies, humic acids and BSA were chosen due to their known fouling propensity and characteristics [1,15]. A 5 L feed water solution was prepared in DI water and mixed with a magnetic stirrer at 350 rpm for 24 h before each experiment to ensure complete dissolution. The feed water was adjusted to a pH of 7 ± 0.5 using H_3_PO_4_, and kaolin clay was added to achieve turbidity of 5 NTU, simulating moderate surface water conditions.

**Figure 1:**
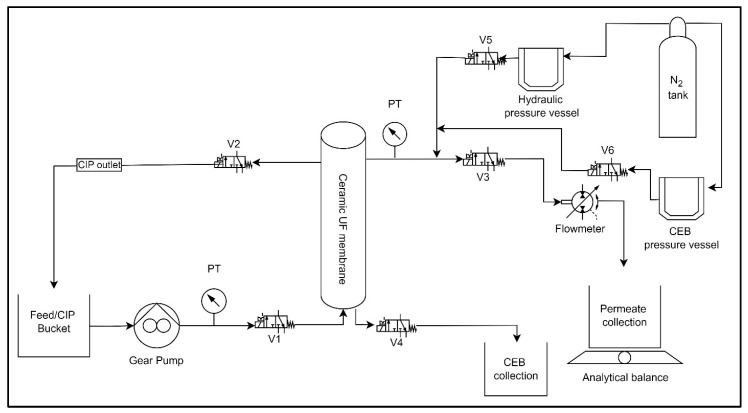
Bench-scale membrane filtration system, where V1 to V6 are the labels for valves to control the flow through the setup. PT stands for pressure transducer.

**Bench-Scale experimental setup and operation:** This study employed the use of an automated ceramic membrane system. Figure 1 displays an illustration of the membrane system, including all the measuring instruments. The system consists of a digital gear pump (Cole Parmer: Drive no. 75211-30, Head no. 07003-04), a flow meter (101 Flo-Sen Mc Millan, 3T), solenoid valves (Macmaster: model no. 4711K731), two pressure transducers (OmegaDyne PX309-100G5V, PX409-030GUSB), and a pressure vessel (Cole Parmer: model no. 29902–90). Nitrogen gas was used to maintain pressure inside the pressure vessel throughout the backwash process. LabVIEW 2015 software (National Instruments, Texas, USA) was programmed to automate system processes. The system regulates the feed pump to maintain a constant flow rate, switches between filtration and backwash cycles, and logs real-time data on key operational parameters such as flow rate, pressure, and mass permeate. This study utilized a ceramic ultrafiltration (UF) membrane with a single channel. The membrane was manufactured by Atech Innovations and consisted of α-aluminum oxide (α-Al_2_O_3_) as the support material and zirconium dioxide (ZrO_2_) as the membrane surface material. Table 1 displays the attributes of the membrane utilized in the research.

The ceramic membrane was fouled with humic acid or BSA in a dead-end flow configuration. An initial flux of 100 L/m^2^h was set for each experiment, which decreases as fouling increases, with data recorded by LabVIEW software. The fouling experiments involved 6 filtration cycles of 30 min with a 60 s backwash at 1 bar pressure after each filtration cycle. The TMP and flux data were logged every 30 s during tests using LabVIEW software. The flux measured during the fouling tests was standardized for temperature using Equation (1).

(1) $$Js={Jm\;(1.03)}^{Ts-Tm}$$

where *Js* and *Jm* represent the standardized and measured fluxes, respectively (L/m^2^h). Ts and Tm are the standard (i.e., 20 °C) and measured temperatures (°C) of the solution, respectively.

Clean water flux (CWF) values were monitored to stabilize baselines and maintain experiment reproducibility. Clean water was filtered through the membrane at flux steps of 100, 120, and 140 L/m^2^h for 1 h, in 20 L/m^2^h increments every 20 min. As the membrane fouls and moves away from the original CWF values, a clean-in-place (CIP) is conducted until it is within 25% of the original conditions. CIP protocol utilized a concentration of NaOH (460 mg/L) and NaOCl (500 mg/L) for 4 h, including two hours of solution recirculation and two hours of soaking [1]. A CWF test was performed between trials to measure the membrane’s permeability (*Jsp*). The standard flux (*Js*) (L/m^2^h) is divided by the differential pressure (Δ*P* in bar) to obtain the clean membrane permeability. *Jsp* was calculated using Equation (2).

(2) $$Jsp=\frac{Js}{∆P}\;$$

**Chemical Cleaning Experiments:** This study examines the combination of surfactant, alkali, and oxidant as a cleaning agent to effectively manage the irreversible fouling of ceramic membranes caused by natural organic matter (NOM), as presented in Table 2. The surfactants utilized in this investigation were Tween 80 and Triton X-100 (Sigma Aldrich, St. Louis, MO, USA). The critical micelle concentration (CMC) values for Tween 80 were reported as 0.015 mM [29] and 0.26 mM Triton X-100 [30]. In addition, the NaOH was obtained from Bioshop with a concentration of 98% *w*/*v*, while the NaOCl was acquired from Lavo (6% *w*/*v*). For CEB, the chemical solutions were prepared in deionized water by mixing a surfactant, an alkali (NaOH), and an oxidant (NaOCl), and for CIP, a mixture of alkali (NaOH) and an oxidant (NaOCl) was prepared in distilled water and utilized.

The combined chemicals contained in the 3-liter stainless-steel tank were subjected to 1 bar pressure using nitrogen gas. The backwash solution was injected into the membrane with an outside-in configuration at regular intervals of 30 min for 60 s, followed by a DI backwash pulse of 30 s to ensure the removal of surfactants from the membrane system. The cleaning efficacy was determined at the end of the experiment.

**Figure 2:**
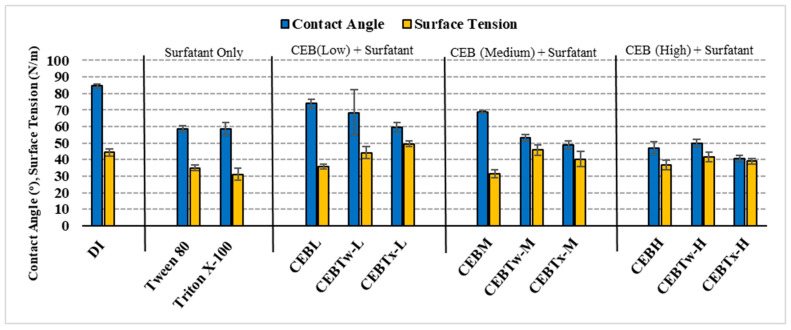
Contact angle (°) and surface tension (mN/m) of different cleaning solutions.

**Contact angle and surface tension measurements:** The wettability of a specific solution was evaluated by measuring the contact angle and surface tension of different cleaning solutions. The measurements were conducted at 22 ± 3 °C, using protocols from [1]. The surface tension (λ) of the cleaning solutions was calculated using Jurin’s law (Equation (3)). Figure 2 displays the contact angle and surface tension measurements of all the solutions.

(3) $$\lambda =\frac{h\times \rho \times r}{2cos(90{}^{\circ}-\theta )}$$

where λ represents the surface tension of a liquid (N/m), *h* is the height of the capillary rise (m), *ρ* is the density of the CEB solution (kg/m^3^), g is the acceleration due to gravity (m/s^2^), *r* is the tube radius (m), and *θ* (theta) is the contact angle of the cleaning solution. The contact angle was determined by measuring it on a flat and smooth hydrophobic surface utilizing VCA Optima equipment. The measurement of capillary rise involved utilizing a slender stem of a clean glass Pasteur pipet; measurements were replicated seven times.

**Carbon Mass Balance:** The carbon mass balance across the membrane was determined by multiplying the total organic carbon (TOC) content (measured in mg/L) of the feed, permeate, and backwash solutions by their respective volumes (L). The efficacy of the cleaning method is determined by the quantity of carbon that remains on the membrane; a lesser mass of carbon indicates a more efficient cleaning process. The quantity of carbon present on the membrane was calculated using Equation (4).

(4) $${TOC}_{Rem}=({TOC}_{feed}\times V_{feed})-({TOC}_{perm}\times V_{perm})-({TOC}_{BW}\times V_{BW})$$

**Membrane Resistance-In-Series (RIS):** To evaluate the membrane fouling, the clean membrane resistance (*K_m_*), reversible fouling resistance (*K_r_*) and irreversible fouling resistance (*K_i_*_r_) were calculated. *K_m_* was derived from Equation (5) where Δ*P* represents the change in transmembrane pressure (bar), *µ* is the dynamic viscosity of water (kg/m·s) and *Js* is the standard flux (typically 20 °C) (L/m^2^h).

(5) $$K_{m}=\frac{∆P}{\mu \times Js}$$

The measured flux and TMP from fouling tests were collected, analyzed, and corrected for temperature (*Js*) (Equation (1)). After temperature adjustment, the resistance in series (RIS) (Equation (5)) model was used to estimate membrane reversible (*K_r_*, m^−1^), irreversible (*K_ir_*, m^−1^), and total fouling resistances. *K_ir_* and *K_r_* were combined to form the total fouling resistance, *K_f_*, where *K_f_* = *K_ir_* + *K_r_*, during each filtration cycle. Thus, the overall resistance was expressed as *K_t_* = *K_m_* + *K_f_* (in m^−1^). Reversible and irreversible fouling resistance is determined by averaging four TMP and *Js* values from the beginning and end of a filtration cycle.

(6) $$Js=\frac{∆P}{\mu (K_{m}+K_{f})}$$

The calculation of the decline of specific flux (%) was performed (Equation (6)) to assess the change in specific flux before and after the filtration test. This calculation employed the specific flux at the beginning of each filtration cycle (*J_sp Beg_*) and the specific flux at the end of the previous filtration cycle (*J_sp End_*) (Equation (7)).

(7) $$Specific\;flux\;decline\;(\%)\;=\;\frac{J_{spBeg}-J_{spEnd}}{J_{spBeg}}\times 100$$

## 3. Results

### 3.1. HA and BSA Fouling on Ceramic UF Membrane

**Figure 3:**
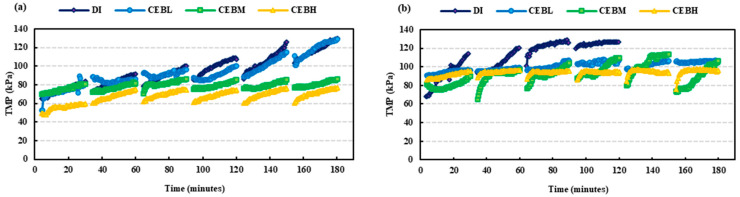
Transmembrane pressure (TMP) over time for (**a**) HA feed and (**b**) BSA feed. The progressive rise in TMP illustrates the accumulation of fouling on the membrane, with higher TMP values indicating increased resistance due to fouling severity.

Filtration experiments were conducted to evaluate the impact of various cleaning solutions on the removal of adsorbed humic acid and BSA protein from the membrane. The fouling and recovery of HA and BSA using NaOH + NaOCl backwash solutions are shown in Figure 3, where transmembrane pressure (TMP) is compared over time. DI (129.7 kPa) and CEB_L_ (129 kPa) exhibited similar variations in TMP throughout the fouling test, whereas CEB_M_ (86.5 kPa) and CEB_H_ (77 kPa) had significantly greater control over fouling, as evidenced by the smaller changes in TMP for HA feed (Figure 3a). The CEB_H_ and CEB_M_ were effective in controlling the fouling due to the higher chemical concentrations and pH. While testing on BSA (Figure 3b), the low, medium, and high concentrations exhibited comparable fouling at 100 kPa; however, the hydraulic backwash exhibited a 25% increase in fouling and remained operational for merely four filtration cycles due to elevated transmembrane pressure and loss of flux, as BSA presents a more pronounced fouling tendency than HA owing to its hydrophilic nature, negative charge, and reduced molecular size. The overall fouling adsorption of HA was lower than BSA, while HA reached a TMP of 120 kPa by the end of cycle 5, BSA with DI water reached 120 kPa at the end of cycle 2.

**Figure 4:**
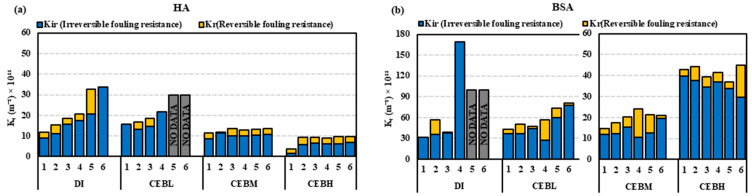
RIS model comparison for (**a**) HA and (**b**) BSA feed with cleaning solutions, higher values indicate greater resistance corresponding to severe fouling. No data indicate the experiment halted due to excessive fouling and membrane pore blockage.

In Figure 4a, the overall fouling, as determined by Resistance-In-Series (RIS), indicates HA fouling resistance (*K_f_*) for CEB_L_ was inefficient with only four filtration cycles, while CEB_M_ and CEB_H_ showed superior fouling control compared to CEB_L_ and DI. Conversely, as seen in Figure 4b, the difference increased significantly for BSA, with DI backwash approaching *K_f_* of 170 × 10^11^ m^−1^ and CEB_L_ to 80 × 10^11^ m^−1^. It was noted that CEB_M_ and CEB_H_ were 8.5 and 4.25 times more in mitigating total fouling due to the higher concentrations that promote stronger interactions with the organic foulants on the membrane surface. CEB_M_, with moderate chemical concentrations, likely allowed more thorough rinsing, minimizing residual accumulation and better fouling removal compared to high concentrations.

Table 3 demonstrated that CEB_L_ (low concentration) was the least effective, showing a notable 50% reduction in specific flux. The TMP increase for both the feeds exhibited an exponential trend (Figure 3), with a 94% increase for HA and an 89% rise for BSA feed during hydraulic (DI) backwash. However, the volume of BSA feed filtered using CEB_L_ was 1295 mL, roughly 50% less than that filtered by CEB_M_ (medium concentration). Conversely, CEB_M_ consistently exhibited enhanced performance. It produced a 60% reduction in specific flux relative to DI water for both HA and BSA. The largest decreases in specific flux were observed for both DI and CEB_L_ solutions for HA and BSA, respectively. Notably, the CEB_M_ appears to have the lowest decline in specific flux for HA and is similar to CEB_H_ for BSA, indicating that an optimum concentration for CEBs may exist when being employed for fouling control. As seen in Table 3, the transmembrane pressure (TMP) rise was regulated efficiently with the BSA feed in comparison to HA. The control resulted from a decrease in flow rate that stabilized the TMP. However, the specific flux data indicated a significant difference in fouling between the two feeds. The volume of permeate collected over the experiments was comparatively higher for HA than BSA, aside from the CEB_M_, suggesting that HA fouling was generally more cake-like in structure while BSA may have exhibited a more pore plugging mechanism [31,32,33].

### 3.2. Removal Efficiency of Surfactant-Enhanced Cleaning Solutions on Ceramic UF Membrane

**Figure 5:**
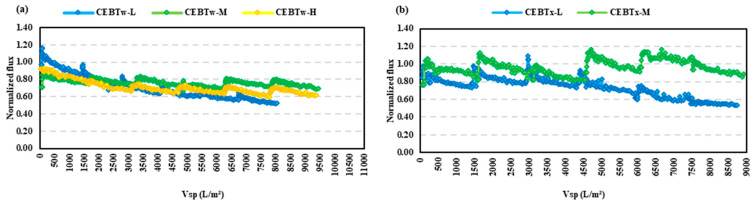
Normalized flux vs. specific volume filtered (Vsp) for HA feed, comparing the impact of varying concentrations with the addition of (**a**) Tween and (**b**) Triton. Vsp is the filtered water volume per membrane area. Experiments were run for equivalent time periods, thus lower Vsp indicates more membrane fouling.

**Figure 6:**
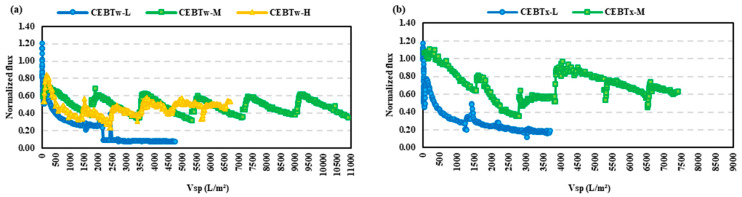
Normalized flux vs. specific volume filtered for BSA feed, comparing the impact of varying concentrations with the addition of (**a**) Tween and (**b**) Triton. Vsp is the filtered water volume per membrane area. Experiments were run for equivalent time periods, thus lower Vsp indicates more membrane fouling.

As the conventional CEB yielded promising results with the initial HA and BSA fouling experiments, this section aims to elucidate further benefits that may be derived from the addition of non-ionic surfactants to the CEB mixture. As shown in Figure 5a, for the HA feed, all concentrations with Tween 80 at CMC performed slightly differently with respect to the fouling control, with CEB_Tw-M_ and CEB_Tw-H_ showing superior performance with the filtered volume of 9500 L/m^2^. The contact angle is an essential parameter in controlling fouling, as it directly indicates the surface wettability of the membrane. The incorporation of Tween 80 into the standard cleaning solution resulted in a 20% reduction in the contact angle for CEB_Tw-M_, indicating a significant improvement in hydrophilicity. This enhanced wettability promotes superior foulant elimination and reduces the adhesion of hydrophobic organic substances. When similar backwash solutions were tested on the BSA feed, a different fouling pattern emerged due to the higher fouling propensity of BSA. Of these, only the CEB_Tw-M_ had the highest specific volume filtered of 11,000 L/m^2^. This is shown in the graph plotted in Figure 6a, where the performance of CEB_Tw-M_ is well-demarcated with respect to the other concentrations. The outstanding performance resulted from the optimal balance between NaOH and NaOCl concentrations, facilitating effective fouling reduction while maintaining membrane integrity. Whereas higher concentrations (CEB_Tw-H_), despite being theoretically more efficient, had a lower performance, because excessive chemical aggression could have caused changes on the surface of the membrane or even formed compact fouling layers [34].

When Tween 80 was substituted with Triton X-100, two distinct concentrations (CEB_Tx-L_ and CEB_Tx-M_) were utilized for both feeds. The experiment on HA feed had a better normalized flux (J’sp) recovery than Tween 80 and when the same was tested on BSA, a drastic difference was seen between the two concentrations, with CEB_Tx-L_ having incomplete filtration with an 80% decline in specific flux. The normalized flux for the HA feed (Figure 5b) indicates that CEB_Tx-M_ was effective in the fouling control, due to their amphiphilic characteristics and hydrophilic (water-attracting) and lipophilic (oil-attracting) properties (HLB), exhibiting only a 15% reduction in normalized flux (J’_sp_) compared to a 47% reduction for CEB_Tx-L_. In Figure 6b, the outcomes for the BSA feed showed significant variance, with a 48% difference for CEB_Tx-M_ and CEB_Tx-L_, alongside a lower volume filtered. Triton X-100 and Tween 80 perform differently due to their HLB ratios and amphiphilic characteristics. The denser micellar layer formed by Triton X-100, with its lower HLB ratio, controlled flux for humic acid and BSA fouling with inconsistency due to the membrane-solution interaction. Tween 80, with its greater HLB ratio and better hydrophilic characteristics, reduced protein fouling (BSA) by disrupting protein-protein and protein-membrane connections.

**Figure 7:**
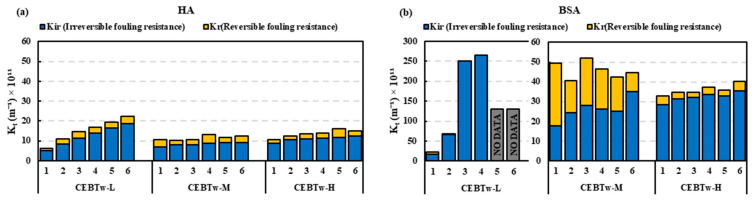
RIS model comparison for (**a**) HA and (**b**) BSA feeds with the addition of surfactants, higher values indicate greater resistance corresponding to severe fouling. No data indicates the experiment halted due to excessive fouling and membrane pore blockage.

Resistance in series (RIS) analysis on the HA feed (Figure 7a) with Tween 80 demonstrated that *K_f_* (Kfouling) values were ≤20 × 10^11^ m^−1^, with CEB_Tw-M_ and CEB_Tw-H_ demonstrating similar resistance patterns with max values nearly 50% of that at CEB_Tw-L_. In comparison, CEB_Tw-L_ (low concentration) backwash was inefficient to control BSA fouling, while CEB_Tw-M_ and CEB_Tw-H_ demonstrated a lower *K_f_* (35 × 10^11^ m^−1^) signifying enhanced backwash efficacy. Figure 7b demonstrates that the resistance analysis corresponded with the normalized flow trends, indicating that CEB_Tw-M_ had a consistent increase in irreversible fouling, although most of the fouling was reversible. Conversely, CEB_Tw-H_ indicated that most of the fouling resistance (*K_f_*) was due to irreversible fouling, underscoring the enhanced cleaning efficacy of CEB_Tw-M_, whereas CEB_Tw-L_ was ineffective in managing fouling under these conditions, as it presented the highest fouling resistance (250 × 10^11^ m^−1^) and the lowest permeate collection.

A 30% specific flux decline was demonstrated (Table 4) for CEB_Tw-M_ on HA feed, while only 6% for CEB_Tx-M_, indicating higher performance with Triton X-100. On the BSA feed, CEB_Tw-M_ had a similar result as HA, with a 24% decline while CEB_Tx-M_ showed a higher flux decline of 40%, which, despite being higher, was far better when compared to the lower concentration (CEB_Tx-L_) that showed a decline of 79%. The higher decline in the flux of CEB_Tx-M_ for BSA is attributed to higher adsorption of protein molecules on the surface due to denser fouling layers compared to humic substances.

### 3.3. Evaluation of Cleaning Efficiency Across Solutions with Equivalent Concentrations

**Figure 8:**
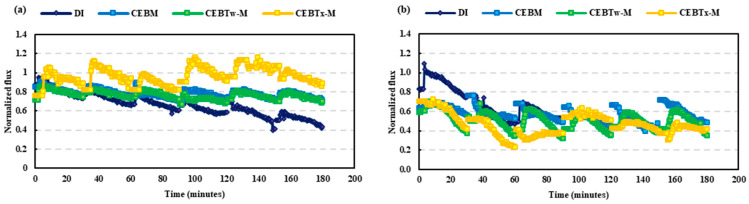
Normalized flux (J’sp) over time, comparing the impact of different backwash solutions on (**a**) HA and (**b**) BSA. The progressive decline in normalized flux illustrates the accumulation of fouling on the membrane, with lower J’sp values indicating increased resistance due to fouling severity.

A comparative analysis of backwash solutions at the mid-level concentrations compared to hydraulic backwash was conducted to confirm their relative efficiency for both HA and BSA feeds. Notably, in all cases, the hydraulic backwash had the lowest recovery performance. In Figure 8, HA CEB_Tx-M_ demonstrated a higher normalized flux control than the Tween-enhanced solution, and the lowest specific flux decline (8%), reflecting its overall efficacy in fouling control. For BSA feed CEB_M_, CEB_Tw-M_, and CEB_Tx-M_ solutions achieved similar normalized flux control, with CEB_Tw-M_ ultimately outperforming all other solutions with a maximum filtered volume of 11,000 L/m^2^ (3372 mL), resulting in a filtration increase of 120% compared to hydraulic backwash. As the filtration cycles advanced, the Tween-enhanced solution (CEB_Tw-M_) efficiently mitigated fouling, ultimately surpassing CEB_M_ and CEB_Tx-M_ with 9000 L/m^2^ (2946 mL) and 7500 L/m^2^ (2267 mL) filtration rates, respectively.

**Figure 9:**
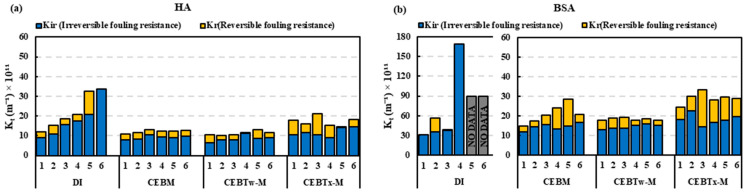
Cleaning efficiency based on reversible and irreversible fouling resistance for various backwash cleaning solutions: (**a**) HA feed (**b**) BSA feed, in comparison to hydraulic backwash. No data indicates the experiment halted due to excessive fouling and membrane pore blockage.

RIS analysis (Figure 9) indicated a superior overall fouling resistance of the Tween 80 enhanced cleaning solution by the end of the trial, relative to other solutions. Significantly, the irreversible fouling of CEB_Tx-M_ during the initial cycle surpassed the levels recorded at the end of the CEB_M_ and CEB_Tw-M_ cycles, suggesting a greater degree of fouling persistence at the beginning of the cleaning process. This suggests that although Triton X-100 might initially manage fouling effectively, its efficacy over an extended period is hindered by significant irreversible fouling. Consequently, CEB_M_ and Tween 80-enhanced solution (CEB_Tw-M_) proved to be an effective solution, exhibiting 70% superior fouling management compared to hydraulic backwash. The higher emulsifying capabilities of Tween 80, along with its gentle effect on membrane surfaces, may have accounted for the maximum specific volume filtered and effective normalized flux regulation [35]. The RIS analysis for BSA (Figure 9) demonstrated that hydraulic backwash is ineffective in managing protein fouling (170 × 10^11^ m^−1^ irreversible fouling), while the incorporation of chemicals like NaOH and NaOCl, which help breakdown organic matter, significantly aids in reducing irreversible fouling (around 20 × 10^11^ m^−1^). Traditional chemical cleaning (CEB_M_) and surfactant-enhanced backwash solutions (CEB_Tw-M_ and CEB_Tx-M_) demonstrated comparable effectiveness in controlling irreversible fouling. In addition, surfactant-based solutions improve cleaning performance, which could enhance membrane longevity and operational efficiency in ultrafiltration systems. Further flux recovery and membrane resistance analysis emphasize the importance of surfactant selection for optimal fouling control.

### 3.4. Reversible and Irreversible Carbon Mass Balance in Wash Waters

**Figure 10:**
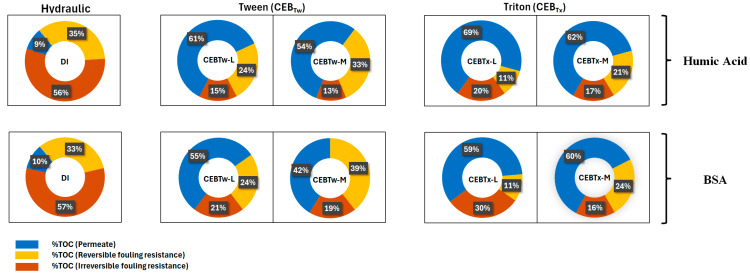
Distribution of carbon mass balance (%) categorized as influent, reversible (carbon in backwash water), irreversible (retained on the membrane), and permeate.

Figure 10 illustrates the carbon mass balance through the different stages of the process: permeate, backwash water (reversible) and carbon remaining on membrane (irreversible). The carbon mass balance through the system revealed a greater proportion of irreversible carbon attached to the membrane surface during hydraulic (DI) backwashes, with 56–57% of total carbon retained on the membrane, in contrast to 13–30% with surfactant + CEB solutions. The results depicted that for both HA and BSA feeds, 40% of the NOM was reversible while no more than 20% was found to be irreversible with Tween 80. Significantly, the BSA feed had a higher proportion of foulants remaining on the membrane compared to the HA feed, which is indicative of higher adsorption of NOM particles and greater irreversibility of fouling further confirmed by the RIS fouling values with BSA. All experiments demonstrated a higher percentage of carbon in the permeate, as expected since ultrafiltration membranes achieve a 25–73% reduction in NOM [35]. This indicates a constraint in NOM rejection, impacting the quality of the permeate. The CEB_Tw-M_ exhibited comparatively better irreversible fouling to CEB_Tw-L_. The cleaning solutions including Triton X-100 exhibit comparable efficiency on HA while had a 50% higher retention of carbon with CEB_Tx-L_ compared to CEB_Tx-M_ on BSA fouling. Triton X-100 enhanced solutions possess elevated carbon levels on the membrane, potentially indicating reduced effectiveness relative to Tween-based cleaning solutions.

Overall Tween 80 CEBs appear to be better performing than Triton X-100 with respect to HA and a substantial difference with BSA, respectively. Carbon mass balance aligns in agreement with other analyses, for instance *K_f_* was < for CEB_Tw-M_ than *K_f_* with CEB_Tx-M_.

## 4. Conclusions

This study evaluated the effectiveness of two commercially available non-ionic surfactants (Tween 80 and Triton X100) in conjunction with traditional cleaning solutions to mitigate NOM fouling in a ceramic ultrafiltration membrane utilizing a chemically enhanced backwash. The results demonstrated that NaOH + NaOCl with and without surfactants could outperform a standard hydraulic backwash. The results further indicate that threshold CEB concentrations may exist where higher concentrations do not improve fouling control, indicating the importance of site-specific testing to determine appropriate CEB solution combinations. Key findings were:

- The lower contact angles of surfactant-enhanced solutions, measuring 53 ± 2° for CEB_Tw-M_ and 48 ± 2° for CEB_Tx-M_, compared to 69 ± 2° for conventional cleaning solution (CEB_M_) and 84 ± 1° for DI water, indicate improved wettability and surface hydrophilicity. Hence, enhancing the cleaning potential by allowing for better penetration and interaction with foulants, resulting in improved fouling control and membrane performance than conventional cleaning solutions and deionized (DI) water.
- The resistance in the series analysis demonstrated that fouling control was most effective in the order of CEB_Tw-M_ > CEB_M_ > CEB_Tx-M_, indicating that CEB_Tw-M_ provided the highest reduction in overall fouling.
- Overall, the irreversibility was lowest for the medium surfactant-enhanced concentrations, where the irreversible fouling retained on membrane with Tween 80-enhanced CEB was 15–21% and Triton X-100-enhanced CEB was 16–30% for HA and BSA as determined by TOC analysis.
- Among all tested cleaning solutions, CEBTw-M exhibited the highest efficacy and reliability in fouling mitigation for both HA and BSA, establishing it as the optimal choice for efficient membrane cleaning in long-term filtration applications; while CEB with surfactants performed better than a hydraulic backwash alone, HA was overall easier to control than BSA.

Additional research with other types of surfactants should be explored as well alternating or staggered hydraulic backwash combined with CEB solutions is recommended to minimize the usage of chemicals required in controlling fouling within membrane systems.

## Figures and Tables

**Table 1:** Characteristics of ceramic UF membrane.

Categories	Contents
Membrane Type	UF ceramic membrane
Material	Ceramic
Support Material	Al_2_O_3_
Surface Material	ZrO_2_
Area	95 cm^2^
Nominal pore size	0.01 µm
Length and Internal diameter	50 cm and 0.6 cm
MWCO	25 kDa

**Table 2:** CEB and CIP solutions with their concentration.

CEB Solution *	Concentration	pH	Abbr.	Feed Solutions
Deionized Water	-	5.4 ± 0.2	-	HA, BSA
NaOH + NaOCl	90 mg/L + 25 mg/L	10 ± 0.2	CEB_L_	HA, BSA
NaOH + NaOCl	230 mg/L + 250 mg/L	10.5 ± 0.2	CEB_M_	HA, BSA
NaOH + NaOCl	460 mg/L + 500 mg/L	12.5 ± 0.2	CEB_H_	HA, BSA
Tween 80 + NaOH + NaOCl	0.015 mM + 90 mg/L + 25 mg/L	11.5 ± 0.2	CEB_Tw-L_	HA, BSA
Tween 80 + NaOH + NaOCl	0.015 mM + 230 mg/L + 250 mg/L	12 ± 0.2	CEB_Tw-M_	HA, BSA
Tween 80 + NaOH + NaOCl	0.015 mM + 460 mg/L + 500 mg/L	12.5 ± 0.2	CEB_Tw-H_	HA, BSA
Triton X-100 + NaOH + NaOCl	0.26 mM + 90 mg/L + 25 mg/L	10 ± 0.2	CEB_Tx-L_	HA, BSA
Triton X-100 + NaOH + NaOCl	0.26 mM + 230 mg/L + 250 mg/L	12.5 ± 0.2	CEB_Tx-M_	HA, BSA
CIP Solutions	Concentration	pH		
NaOH + NaOCl	460 mg/L + 500 mg/L	12.1 ± 0.2	-	

* NaOCl is reported as mg/L of Cl_2_; HA = humic acid, BSA = bovine albumin serum.

**Table 3:** Volume filtered, SFR, and TMP with cleaning solution and foulant type.

Backwash	TMP (kPa)	*J_sp Beg_* (L/m^2^h /KPa)	*J_sp End_* (L/m^2^h /KPa)	Volume Permeate Collected (mL)	%Spec Flux Decline	%TMP Rise
**Humic Acid (HA)**						
**DI**	127.7	1.439	0.66	2795	54	94
**CEB_L_**	127.6	1.379	0.689	2916	50	85
**CEB_M_**	85.1	1.407	1.122	2868	20	23
**CEB_H_**	76.5	1.814	1.224	2807	33	55
**Protein (BSA)**						
**DI**	128.9	1.359	0.284	1531	79	89
**CEB_L_**	106.1	0.93	0.4	1640	57	35
**CEB_M_**	104.0	1.234	0.884	2946	28	32
**CEB_H_**	96.8	0.776	0.563	2251	27	11

**Table 4:** Volume filtered, SFR, and TMP for surfactant-enhanced cleaning solutions.

Backwash Composition	TMP (kPa)	Jsp Beg (LMH/kPa)	Jsp End (LMH/kPa)	Volume Permeate Collected (mL)	%Spec Flux Decline	%TMP Rise
**Humic Acid (HA)**						
***Tween 80 + NaOH + NaOCl (CEB)***						
**CEB_LTw_**	100.00	1.379	***0.689***	2498	50	67
**CEB_MTw_**	83.77	1.618	1.127	2881	30	21
**CEB_HTw_**	85.77	1.353	1.121	2984	17	43
***Triton X100 + NaOH + NaOCl (CEB)***						
**CEB_LTx_**	100.66	1.297	0.793	2734	39	43
**CEB_MTx_**	88.46	0.948	0.894	2856	6	8
**Protein (BSA)**						
***Tween 80 + NaOH + NaOCl (CEB)***						
**CEB_LTw_**	131.00	1.156	0.127	1299	89	58
**CEB_MTw_**	103.42	1.048	0.793	3372	24	15
**CEB_HTw_**	95.22	0.92	0.515	2023	44	15
***Triton X100 + NaOH + NaOCl (CEB)***						
**CEB_LTx_**	116.87	1.036	0.213	1414	79	32
**CEB_MTx_**	95.91	1.216	0.727	2267	40	29

## Data Availability

Data are contained within the article.
